# Fungal and bacterial diversity of Svalbard subglacial ice

**DOI:** 10.1038/s41598-019-56290-5

**Published:** 2019-12-27

**Authors:** L. Perini, C. Gostinčar, N. Gunde-Cimerman

**Affiliations:** 1 Department of Biology, Biotechnical Faculty, University of Ljubljana, Jamnikarjeva 101, SI-1000 Ljubljana, Slovenia; 2 Lars Bolund Institute of Regenerative Medicine, BGI-Qingdao, Qingdao, 266555 China

**Keywords:** Biodiversity, Microbial ecology

## Abstract

The composition of fungal and bacterial communities in three polythermal glaciers and associated aquatic environments in Kongsfjorden, Svalbard was analysed using a combination of cultivation and amplicon sequencing. 109 fungal strains belonging to 30 mostly basidiomycetous species were isolated from glacial samples with counts up to 10^3^ CFU/100 ml. *Glaciozyma*-related taxon and *Phenoliferia psychrophenolica* were the dominant species. Unexpectedly, amplicon sequencing uncovered sequences of Chytridiomycota in all samples and Rozellomycota in sea water, lake water, and tap water. Sequences of *Malassezia restricta* and of the extremely halotolerant *Hortaea werneckii* were also found in subglacial habitats for the first time. Overall, the fungal communities within a glacier and among glaciers were diverse and spatially heterogenous. Contrary to this, there was a large overlap between the bacterial communities of different glaciers, with *Flavobacterium* sp. being the most frequently isolated. In amplicon sequencing Actinobacteria and Proteobacteria sequences were the most abundant.

## Introduction

The archipelago of Svalbard is located in the Arctic Ocean between Norway and the North Pole. It is one of the best studied locations in the Arctics and thus considered a “hot spot” of Arctic microbiological research. So far, studies of microbial glacial communities have focused on either Bacteria^1–3^, Fungi^4–7^, Archaea^8^, or viruses^9,10^. Analyses of more than one of these groups from the same samples and particularly the investigation of fungal diversity with molecular methods have not been published so far.

Glaciers with a polythermal basal regime^11^ are defined by the presence of water at the subglacial drainage system at their basis^12^. They are entirely warm-based, except at the margins, meaning that liquid water present at the glacier bed is generated by surface flow, friction, pressure melting, and groundwater infiltration^13^. This base consists of a relatively thin layer of debris-rich ice called subglacial ice or basal ice formed by subsequent freezing and melting of subglacial waters and entrainment of subglacial sediment and debris^14,15^. For a long time these environments were considered abiotic, but later research has shown that they represent an important share of global microbiological activity that has received some attention only in the last two decades^13,16–19^. A few years after the first systematic investigations of subglacial bacteria, fungi were also found to be able to survive in these extreme environments, exemplified by large populations of *Penicillium* spp.^6^, basidiomycetous^4^ and ascomycetous yeasts^20^.

**Figure 1:**
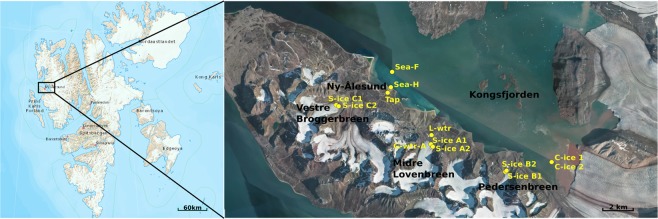
Location of the sampling site in the Kongsfjorden area, with insert showing the relative position of the sampling region within Svalbard. Map is acquired as an image from TopoSvalbard credit NPI/USGS Landsat, with courtesy of the Norwegian Polar Institute. Norwegian Polar Institute. *Svalbard map* [map]. Scale [1:6000000]. “TopoSvalbard” <https://toposvalbard.npolar.no> (Accessed January 21, 2019); *Map data and orthophoto based on aerial imagery of 2010 and 13828* [map]. Scale [1:200000]. “TopoSvalbard”<https://toposvalbard.npolar.no>(Accessed January 21, 2019).

This report focuses on three polythermal Svalbard glaciers located on the southern side of Kongsfjorden (Fig. 1) near Ny-Ålesund (“breen” is glacier in Norwegian): Midtre Lovénbreen, Pedersenbreen, and Vestre Brøggerbreen^21–24^. So far all studies of the microbial communities of the subglacial environments of these glaciers were based on cultivation. Here we focused on fungal and bacterial communities found in the subglacial ice of these glaciers and in the associated aquatic environments such as clear ice floating in sea water, glacial meltwater, moraine lake water, sea water and lake-derived tap water. The microbial communities were investigated using traditional culturing and, for the first time in case of subglacial ice fungi, high-throughput amplicon sequencing methods. Our findings show a considerable diversity (particularly for fungi) amongst local microbial communities and include the presence of a number species not expected in the Arctic environment.

## Results

Fungal and bacterial diversity of subglacial ice of three polythermal glaciers and their surrounding aquatic environments were analysed by a combination of cultivation and molecular methods.

### Quantification of cultivable fungi in subglacial ice and glacial meltwater displayed major differences between glaciers and within glaciers

In cultivation experiments using several different media on which the filtered samples were inoculated, fungal counts differed considerably depending on the media used **(**Supplementary Material**)**. Overall the lowest counts were obtained on the two low a_w_ media, on MY10–12 only up to 88 CFU/100 ml, while on DG18 they ranged from 4.8 CFU/100 ml up to 500 CFU/100 ml. On the enumeration medium DRBC the highest counts were somewhat higher, from 0 to 540 CFU/100 ml. Highest counts were obtained on two low-nutrient media, where up to 1900 CFU/100 ml (MM) and up to 2400 CFU/100 ml (SNA) were detected.

Overall, subglacial ice and glacial meltwater counts were comparable between samples. Glacier Pedersenbreen had both the highest (in sample S-ice B2) and the lowest (in the sample S-ice B1) fungal abundance. S-ice B2 counts were up to 2400 CFU/100 ml, while counts for S-ice B1 did not exceed 37 CFU/100 ml. Vestre Brøggerbreen samples (S-ice C1 and S-ice C2) also differed considerably in fungal abundance. While in S-ice C1 they ranged from 45 to 990 CFU/100 ml (MM), in S-ice C2 they were in the range from 5 to 200 CFU/100 ml. Values obtained for Midtre Lovénbreen (S-ice A1, S-ice A2) were similar between the samples, and ranged from 0 to 120 and 72 CFU/100 ml, respectively.

No CFU were observed for some glacial meltwater (G-wtr A) samples on the low a_w_ media, but on oligotrophic media the number of fungi reached up to 530 CFU/100 ml.

### Fungal diversity (amplicon sequencing) revealed dominance of different basidiomycetous fungi

In total 3,938,443 paired-end reads were obtained from 12 samples. The reads corresponded to 740 different operational taxonomic units (OTUs). The number of reads per sample ranged from 174,770 (L-wtr) to 503,852 (S-ice-C1). Shannon indices ranged from H′ = 1.25 (S-ice-C1) to H′ = 3.63 (S-ice-B2), revealing high differences in diversity between the samples. Rarefaction curves suggested that Illumina sequencing depth was sufficient to capture the dominant phylotypes in all samples (Supplementary Material). To test for differences in the fungal taxa as determined by QIIME2 between sample types PERMANOVA multivariate diversity analyses were performed. Significant overall differences were found between the three major types of samples (i.e., subglacial ice, clear ice, sea water, pseudo-F = 4.54; p = 0.002; the number of permutations = 999). However, the pairwise differences between the sample types as analysed by PERMANOVA were not statistically significant, possibly due to the small numbers of samples of each sample type.

**Figure 2:**
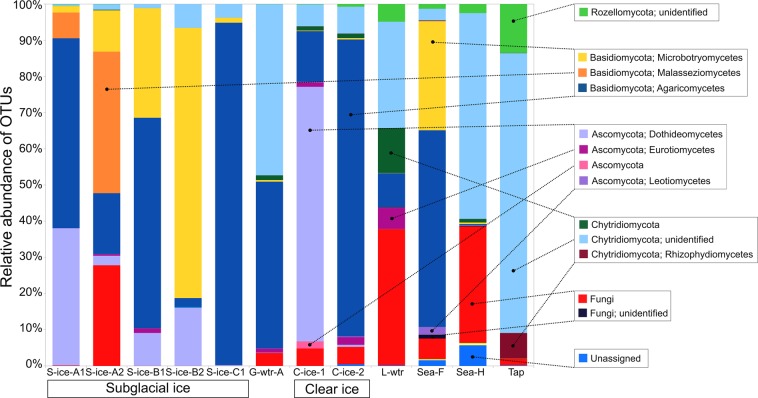
Percent of fungal operational taxonomic units (OTUs) aligned and assigned to known fungal classes based on PCR amplifications of ITS2 gene sequences for all the sample types.

Although subglacial ice samples differed substantially in their diversity both between and within the glaciers, they were nevertheless all dominated by Basidiomycota (from 61% in S-ice-A1 to 96% in S-ice-C1), with Microbotryomycetes and Agaricomycetes being the most abundant classes of fungi (Fig. 2). Also, all subglacial ice samples contained at least some sequences representing Chytridiomycota (from 0.4% in S-ice-A1 to 7% in S-ice-B2).

Vestre Brøggerbreen glacier (S-ice-C1) was totally dominated by Agaricomycetes (95%), primarily the family Schizoporacea (90% sequences), while Pedersenbreen glacier S-ice-B2 was dominated by Microbotryomycetes, with a relative frequency of 75%. A large part of these sequences were placed in the order Leucosporidiales (53%), present in lower abundance also in S-ice-B1, S-ice-A2, S-ice-C1. The order Pleosporales was present in S-ice-B2 (16%), S-ice-B1 (9%), and S-ice-A2 (2%).

Both Midtre Lovénbreen glacier samples were substantially different the other two glaciers. S-ice-A1 surprisingly contained an abundance of sequences identified as the ascomycetous black yeast *Hortaea werneckii* (Dothideomycetes class, 38%), and the giant polypore *Meripilus giganteus* (Agaricomycetes class, 62%) (Fig. 2). The latter species also dominated in S-ice-B1 of Pedersenbreen glacier (47%), sea fjord (54%), and glacial meltwater (45%). The other Midtre Lovénbreen sample, S-ice-A2, differed from other samples in the high presence of *Malassezia restricta* (Malasseziomycetes class, 39%) and a large proportion of unassigned fungi (28%).

Clear ice samples were again very different from one another: C-ice-1 was dominated by Ascomycota (74%), the class Dothideomycetes (70%), primarily represented by the black yeast of the orders Dothideales (21%) and Capnodiales (15%). On the other hand C-ice-2 was characterised by the species *Schizopora flavipora* (81%) belonging to the class Agaricomycetes, unidentified Chytridiomycota (7%) and unidentified species (5%).

**Figure 3:**
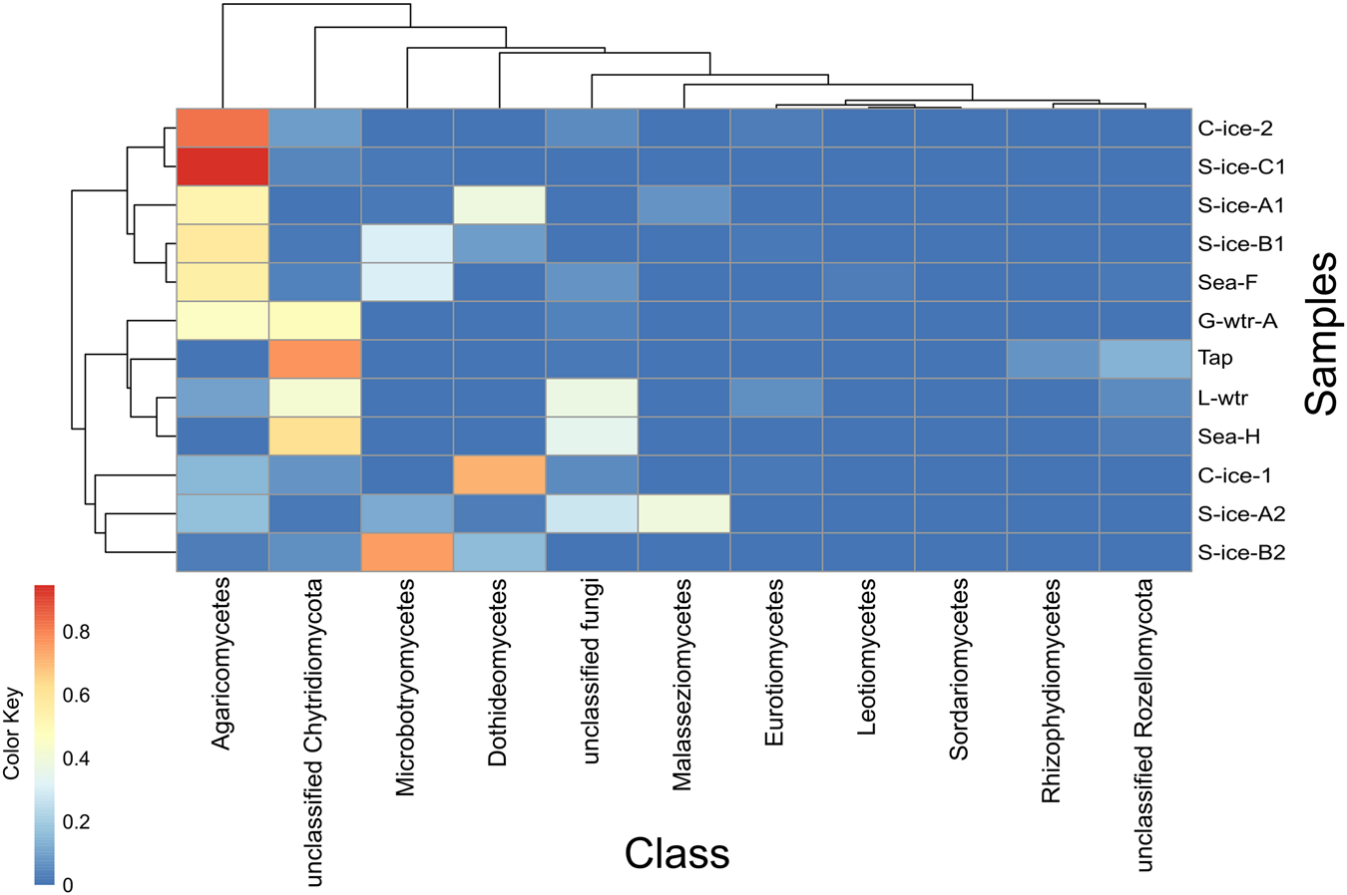
Heatmap showing the total abundance of each class in each sample of subglacial ice (S-ice), clear ice (C-ice), sea water (Sea), glacial meltwater (G-wtr), lake water (L-wtr), and tap water (Tap) collected from Ny-Ålesund, Svalbard. The branch length of the cluster dendogram show the similarity level. Clustering among samples was based on hierarchical cluster analysis (hclust).

Moraine lake water, glacial meltwater, harbour sea water, and indoor tap water were largely characterised by unidentified Chytridiomycota (42%, 48%, 57%, and 84%, respectively) (Fig. 3). Phylum Rozellomycota was identified in Ny-Ålesund harbour sea water, moraine lake water and tap water (3%, 5%, and 13%, respectively).

Core microbiome analysis showed that 28 OTUs were shared between all subglacial ice samples, of which 13 belonged to Basidiomycota, 7 to Chytridiomycota, 5 to Ascomycota, and 3 to unassigned Fungi. *Hortaea werneckii* and *Malassezia restricta* were among the species present in all the subglacial samples.

**Figure 4:**
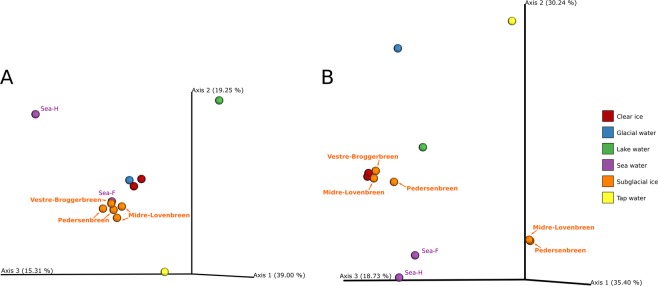
Principal coordinate analysis (PCoA) ordination patterns based on weighted uniFrac distance of fungi (**A**) and bacteria (**B**).

The principal component analyses (PCoA) of ordination patterns revealed four separate sample clusters based on phylogenetic distance metrics (weighted uniFrac distance) (Fig. 4A). The three axes of PCoA accounted for 74% of the variation. All subglacial ice samples clustered with sea water sampled in the fjord, and were also relatively close to clear ice samples, and glacial meltwater. Tap water, harbour sea water and lake water were, according to this analysis, distinct in their fungal diversity.

### Cultivable fungal diversity in subglacial ice and glacial meltwater showed major differences within and between glaciers

From subglacial ice and glacial meltwater, 113 fungal isolates were obtained, belonging to 19 genera and 30 different species of filamentous fungi and 10 genera and 11 species of yeasts. Vestre Brøggerbreen S-ice C1 sample harboured the most diverse fungal community that contributed the most to the overall dissimilarity, as confirmed by SIMPER analysis (Supplementary Material). The most frequently isolated yeasts were basidiomycetous *Glaciozyma*-related taxon, *Mrakia* sp., *Glaciozyma watsonii*, and *Rhodotorula svalbardensis* pro. tem. and more sporadically an unidentified basidiomycete. Amongst filamentous fungi *Pseudogymnoascus* sp., and *Penicillium bialowiezense* prevailed. Less frequent were *Botrytis cinerea*, *Cladosporium* sp., *Geomyces* sp., unidentified *Helotiales* sp. 2, *Leptosphaeria* sp., *Mortierella* sp., *Penicillium swiecickii*, *Phialocephala* sp., *Pseudeurotium* sp., *P. hygrophilum*, *Tetracladium* sp. and *Thelebolus* sp. The other Vestre Brøggerbreen sample, S-ice C2, displayed in comparison a much lower diversity. Isolates were identified as *Glaciozyma*-related taxon, *Pseudogymnoascus* sp., unidentified *Helotiales*, sp. 1 and unidentified Ascomycota sp. 4.

Midtre Lovénbreen glacier (S-ice A1 and S-ice A2) samples were dominated by yeasts. In S-ice A1 *Glaciozyma*-related taxon isolates prevailed, followed by *Phenoliferia psychrophenolica*, *Rhodotorula svalbardensis* pro. tem., and Basidiomycota sp. 2^25^. Filamentous fungi were represented by unidentified *Helotiales* sp. 1, *Lecanicillium* sp., unidentified Ascomycota sp. 2, and unidentified Ascomycota sp. 3. S-ice A2 was again dominated by yeasts: *Filobasidium magnum*, *Naganishia albida*, and *Rhodotorula svalbardensis* pro. tem., *Phenoliferia psychrophenolica*, unidentified Ascomycota sp. 1, filamentous *Cladosporium pseudochalastosporoides* and unidentified Venturiaceae.

The two subglacial ice samples (S-ice B1 and S-ice B2) of Pedersenbreen glacier were again strongly dissimilar. In S-ice B1 penicillia (12 CFU/100 ml) prevailed, with *Penicillium crustosum* as the dominant species and occasional isolates of *Venturia* sp. The most frequently isolated yeast was *Glaciozyma*-related taxon, followed by *Filobasidium magnum, Phenoliferia psychrophenolica*, *Ph. glacialis* and *Sporidiobolus* sp. S-ice B2 had the highest yeast abundance (500 CFU/100 ml) (Supplementary Material) and was characterized in particular by *Glaciozyma*-related taxon, *Glaciozyma watsonii*, *Mrakia* sp. and *Phenoliferia psychrophenolica*. More sporadic yeasts/fungi were *Leucosporidiella muscorum*, *Vishniacozyma victoriae*, unidentified Ascomycota sp. 1, unidentified *Helotiales* sp. 3, and *Paraleptosphaeria dryadis*.

Glacial meltwater (G-wtr A) enviro Filamentous fungi were represented by unidentified nment harboured primarily unidentified *Helotiales* sp. 2, sporadic basidiomycetous yeasts *Glaciozyma*-related taxon, *Glaciozyma watsonii*, *Mrakia* sp. and *Phenoliferia psychrophenolica* and filamentous fungi *Collybia cirrhata* sp., and *Venturia* sp.

Analysis of the shared diversity (comparison between ITS2 sequences extracted from culture-sourced ITS sequences and from the amplicon sequencing) between the cultivation and the amplicon sequencing dataset showed that out of 30 species found by cultivation, 8 were also found by amplicon sequencing (27% of the diversity found with both approaches) (Supplementary Material). These species were *Penicillium crustosum*, unidentified Basidiomycota closely related to *Peniophora* sp., *Cladosporium* sp. and *Cladosporium pseudochalastosporoides*, Helotiales sp. 3, *Lecanicillium* sp., *R. svalbardensis* pro. tem., and *Thelebolus* sp.

### Subglacial ice and glacial meltwater have similar abundances of cultivable bacteria

Counts of bacteria on R2A medium were calculated (Supplementary Material). Overall, subglacial ice and glacial meltwater samples had similar bacterial counts, with values spanning from 300 CFU/100 ml (S-ice B1) up to 3300 CFU/100 ml (S-ice B2).

### Diversity of bacteria (amplicon sequencing) revealed a prevalence of Actinobacteria and Proteobacteria

After subtraction of chloroplast sequences, the dataset was composed of 1,793,737 assembled sequences (for 12 samples in total), corresponding to 712 different features OTUs. Number of reads per sample ranged from 280,884 (C-ice-2) to 62,627 (Sea-H). Shannon indices for bacteria varied over a broad range from H′ = 2.5 (C-ice-1) to H′ = 5.5 (Sea-F) indicating large differences in the diversity of individual samples. Samples with lower bacterial diversity indices were both samples of clear ice (C-ice-1, C-ice-2), as well as four of the six subglacial ice samples (S-ice-A1 and A2, S-ice-B2 and S-ice-C1). Nevertheless, significant differences were found between all three types of samples (i.e., subglacial ice, clear ice, sea water, pseudo-F = 4.54; p = 0.012; the number of permutations = 999). However, no statistically significant differences were found in the pairwise PERMANOVA comparisons of sample types. Rarefaction curves suggested that Illumina sequencing depth was sufficient to capture the dominant phylotypes in all samples (Supplementary Material).

**Figure 5:**
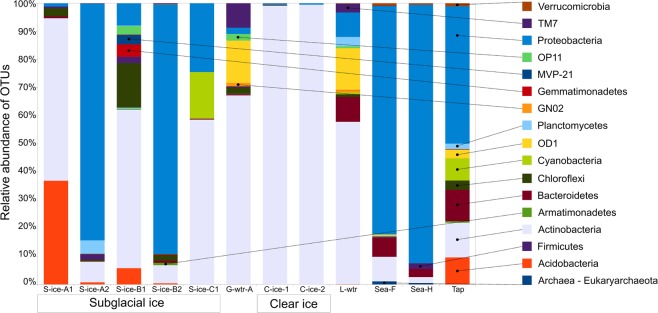
Percent of bacterial operational taxonomic units (OTUs) aligned and assigned to known bacterial phyla based on PCR amplifications of 16S gene sequences for all the sample types.

The common bacterial phyla across all 12 samples were Actinobacteria and Proteobacteria, with wide ranges of relative abundances (99–2%, 92–0.5%, respectively) (Fig. 5).

Midtre Lovénbreen subglacial ice samples were characterised by the above-mentioned phyla, but in different proportions. In S-ice-A1 Actinobacteria (genus *Micrococcus*) was present at up to 58%, while Proteobacteria reached only 1% (alpha-proteobacteria - Sphingomonadetes). Other sequences belong to Acidobacteria (37%) and Chloroflexi (3%). In S-ice-A2 Proteobacteria (epsilon-proteobacteria - Campylobacteriales) were the most abundant, followed by Actinobacteria (7%), Planctomycetes (order Gemmatales – 5%) and Firmicutes (2%). The phylum Planctomycetes was additionally identified in lake water (3%) and tap water (2%).

Although Pedersenbreen glacier, samples S-ice-B1 and S-ice-B2 shared some taxa, they were present with different frequencies: Actinobacteria (56 and 6%, respectively), Proteobacteria (8 and 89%, respectively), Chloroflexi (16 and 2%, respectively). Additionally S-ice-B1 contained Acidobacteria (6%) and Gemmatimonadetes (4%).

The only sample from Vestre Broggerbreen glacier, S-ice-C1, was dominated by Actinobacteria (genus *Microccocus*, 57%), followed by Proteobacteria (alpha-proteobacteria – Sphingomonadales, 24%) and Cyanobacteria (17%).

Clear ice samples were entirely dominated by Actinobacteria (genus *Micrococcus*) with frequencies higher than 99%. In glacial meltwater Actinobacteria sequences prevailed with a relative abundance of 67%, but it contained additional characteristic taxa, such as OD1 (15%), TM7 (9%) and OP11 (2%). Lake water also contained phylum OD1 (15%), together with Bacteroidetes (9%), Proteobacteria (9%) and Actinobacteria (58%). Sea water samples, Sea-F and Sea-H, were dominated by Proteobacteria (alpha-proteobacteria, 81 and 92%, respectively). Tap water was dominated by the phylum Proteobacteria (49%), followed by Actinobacteria (12%), Bacteroidetes (11%), Cyanobacteria (8%), Acidobacteria (10%) and Chloroflexi (3%).

The principal component analyses (PCoA) of ordination patterns revealed five separate sample clusters based on phylogenetic distance metrics (weighted uniFrac distance) (Fig. 4B). The three axes of the PCoA accounted for 84% of the variation. Samples of subglacial ice (S-ice-A1, S-ice-B1 and S-ice-C1), clear ice and lake water clustered together. The second cluster was composed of the two subglacial ice samples S-ice-A2 and B2 that shared a very similar bacterial composition. Sea water samples formed the third cluster, while glacial water and tap water were clearly separated from all the other samples.

### Diversity of cultivable bacteria reflected differences observed with NGS

52 bacterial isolates were obtained from subglacial ice and glacial meltwater samples. In Midtre Lovénbreen all isolates from S-ice A1 belong to *Flavobacterium* sp., while S-ice A2 showed the highest bacterial subglacial diversity. Although *Flavobacterium* sp. again prevailed, isolates belonging to *Cryobacterium* sp., *Hymenobacter* sp., *Massilia* sp., *Polaromonas* sp., *Pseudomonas graminis*, *Spirosoma* sp., and an uncultured species belonging to Oxalobacteraceae family, were also retrieved.

In Pedersenbreen glacier a similar pattern was observed. Sample S-ice B2 was monopolized by *Flavobacterium* sp., while S-ice B1 additionally harboured *Cryobacterium* sp., *Massilia* sp., *Paenibacillus antarcticus* and an uncultured species belonging to Oxalobacteraceae family, closely related to *Actimicrobium antarcticum*.

Vestre Brøggerbreen S-ice C1 isolates were identified as *Cryobacterium* sp., *Massilia* sp., and *Pseudomonas* sp., while S-ice C2 contained as *Flavobacterium* sp., *Herminiimonas* sp., and unidentified Oxalobacteraceae clustering with S-ice A2 isolate.

Glacial meltwater had the most diverse cultivable bacterial community. The most frequently isolated species were *Pseudomonas* sp. (including *P. frederiksbergensis* and *P. graminis*), followed by *Flavobacterium* sp., and *Massilia* sp. Among sporadically isolated species were *Polaromonas* sp., *Sphingomonas* sp., unidentified Burkholderiaceae, and *Sphingorhabdus* sp.

## Discussion

Glacier ice is one of the most challenging natural environments for life. Nevertheless, glacial habitats harbour a wide diversity of microorganisms, both prokaryotes and eukaryotes. Some microbes deposited on the glacial surface gradually travel to deeper ice layers. They can survive in ice as viable, frozen “living fossils”^26,27^, while others remain metabolically active and possibly multiply in the veins of brine between ice crystals^28^. Microbial communities also develop in deep ice or subglacial ice at the base of polythermal glaciers, where melting occurs due to gravitational pressure resulting in thin layers of liquid water, often with high concentrations of salts and enriched with the inorganic material of the glacier bed. These glacial populations likely represent an important share of global biological activity^13,16–19^ that has so far been poorly characterized. A deeper understanding of the microbial biodiversity of this unique and rapidly disappearing ecosystem would contribute to our understanding of the composition and dynamics of subglacial microbial communities and their roles in the Arctic ecosystems. Therefore, fungal and bacterial diversity of oligotrophic subglacial and glacial ice and meltwater and nearby marine water, moraine lake water and tap water were investigated both by cultivation and with molecular methods, the latter being applied on samples of subglacial ice for the first time.

Fungi were abundant in all samples as shown by both cultivation and amplicon sequencing. Frequencies in the three glaciers differed considerably. Midtre Lovénbreen harboured the lowest number of cultivable fungi (up to 100 CFU/100 ml), followed by Vestre Brøggerbreen (up to 990 CFU/100 ml) and Pedersenbreen (1000 CFU/100 ml) including yeasts at 500 CFU/100 ml) (Supplementary Material). The large proportion of yeast in Pedersenbreen was confirmed by amplicon sequencing, where the highest number of sequences belonged to Microbotryomycetes (75%). Sixty-eight % of the cultured yeast species belonged to the Microbotryomycetes and the remaining 32% to the class Tremellomycetes (although the latter were absent in amplicon sequencing results). A previous cultivation study^4^ reported even higher yeast counts in Svalbard glacial ice (up to 4000 CFU/ml), while Turchetti *et al*.^29^ reported similar counts in Alpine subglacial sediments (100 to 1000 CFU/g). Both studies reported several orders of magnitude lower numbers of yeast cells in supraglacial ice and sediments indicating an enrichment of yeast in subglacial environments. Such enrichment could be supported by the presence of organic carbon in subglacial sediments^17^, deriving from permafrost soils that are overridden by the advancing glaciers and finely ground by subglacial abrasion processes. This organic material consists of cyanobacterial mats, plant material, and roots, which are readily biodegradable by microbial activity^17^.

**Figure 6:**
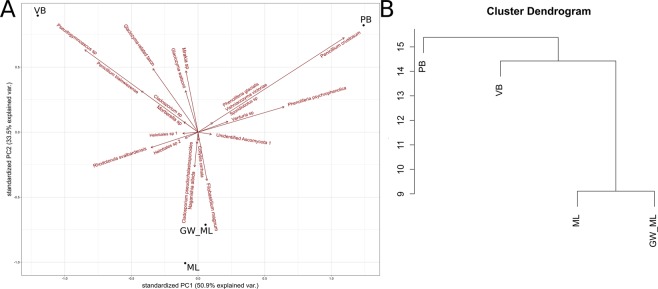
Principal Component Analysis (PCA) based on presence/absence data of fungal species in the studied environments. Abbreviations: Midtre Lovénbreen (ML); Midtre Lovénbreen glacial meltwater (GW_ML); Vestre Brøggerbreen (VB); Pedersenbreen (PB).

Our results show a significant spatial variability of fungal communities within individual glaciers as well as between glaciers, suggesting/highlighting that the microbial diversity in glacial ice is much more than just a collection of deposited propagules. Many species were recovered from only one of the two samples of the same glacier (Fig. 6A) indicating a heterogeneous nature of the glacial habitats, a phenomenon already described by Luo *et al*.^30^. In contrast, meltwater flowing from under the glacier was very similar in fungal composition to the nearby subglacial ice sample (cf. the hierarchical clustering dendogram of Midtre Lovénbreen glacier (Fig. 6B)). Differences in the microbial diversity might be explained by the fact that basal ice is composed of individual layers of ice with physico-chemical heterogeneity favouring the establishment of microhabitats^14^. Basal ice microbiota is not only influenced by the Aeolian inoculum deposition at the surface, but also by its anisotropic structure at the bottom. Spatial and/or temporary differences in deposition together with environmental filters acting on the resident microbiota can be envisaged to lead to a patchy distribution of species within the glacier and at the same time point to a low amount of microbial migration within the glacier, which would instead result in a homogenous distribution of species.

Analysis of the ITS2 amplicon discovered sequences assigned to the phylum Chytridiomycota in all samples, with the highest abundance in tap water, followed by harbour sea water, lake water, and glacial meltwater. In the past isolations of chytrids from extreme habitats were rare. However, recent metagenomic studies revealed their widespread occurrence in a broad range of extreme habitats such as snow in Europe and America^31^, Antarctic soils^32,33^, Patagonian glacier ice, meltwater and sediments^34,35^. In Arctic marine environments they are even the dominant fungi^36–41^. This is to the best of our knowledge the first report of Chytridiomycota in Arctic glacial ice and tap water. Chytridiomycota in household tap water probably originate from the water source itself, which in case of Ny-Ålesund water supply system is a surface lake filled by the snow meltwater^42^, and are possibly enriched during transportation to the final user. Tap water and to a lesser extent moraine lake water (from a different lake than the one providing the tap water) were also populated by unidentified Rozellomycota, an early diverging hyper-diverse lineage of fungi also known as Cryptomycota^43^ that have been documented almost exclusively by NGS analyses of environmental samples^44^. These uncharacterized fungi dominate fungal communities in different aquatic ecosystems, such as temperate freshwater lakes^45–48^, coastal and marine environments^49–51^, snow^31^ and polar aquatic systems^36,39,52,53^. Their abundant presence in glacial and related samples is in agreement with these findings, since the nature of samples analysed in this study is also aquatic, and/or is possibly linked to the ability of Rozellomycota to mycoparasitise chytrids^54,55^.

The amplicon sequencing showed strong dissimilarities between samples obtained from the same glacier and between glaciers, an observation also confirmed by cultivation. For instance, *Libkindia masarykiana* was abundantly present in the sample S-ice-B2, but was present in very low numbers in the other samples. C-ice-1 was characterized by Dothideomycetes, while C-ice-2 was dominated by *Schizopora flavipora* otherwise known as a tea plant pathogen. Also, fungi in the sea water sampled in the harbour were different from the seawater fungi sampled in the middle of fjord (which was more closely related to subglacial ice samples (Fig. 4A)).

Among the most unexpected results of our study was the presence (as shown by NGS) of the black yeast *Hortaea werneckii* (38%) in Lovenbreen glacier (S-ice-A1), never reported before in any glacial environment. The fungus was present also in all the other subglacial samples, but in lower frequencies. This extremely halotolerant black yeast is the dominant fungus in hypersaline waters of salterns worldwide^56^, and is additionally found in other marine related environments (e.g. surface sea water, sea sponges, corals, fish, halophites)^57–59^ and deep sea^60–62^. Furthermore, *H. werneckii* is known as the etiological agent of human *tinea nigra*, a superficial infection of salty soles and palms in humans, occurring in tropical and subtropical areas^63,64^.

The second sample of Lovenbreen glacier (S-ice-A2) was characterized by the presence of *Malassezia restricta* (39%), the most common human skin-related fungus after *M. globosa*^65–67^. Initially *Malassezia* spp. were thought to be specifically associated with mammalian hosts, but culture-independent studies revealed their presence in very diverse marine and terrestrial ecosystems, such as cone snails^68^, Antarctic soils^69,70^, deep-sea sediments^71^, deep-sea hydrothermal vents^61,72^, nematodes^73,74^, corals^75^, and sponges^76^. Amend^72^ found that ribosomal DNA sequences of *Malassezia* in the above-cited studies are nearly identical to sequences of human-associated isolates, suggesting that they either diverged in their habitats very recently or that they are capable of large adaptability and high stress tolerance enabling them to colonize environments as different as human skin and subglacial ice. The fact that this species was not detected by cultivation may be due to its slow and fastidious growth and lipophilic nature, usually not taken into consideration in standard growth media^77^.

Cultivation of various samples confirmed some of the similarities between sample sites as observed by NGS: the most frequently isolated yeast species belonged to the basidiomycetous *Phenoliferia psychrophenolica* (former *Rhodotorula psychrophenolica*) and were similar to *Glaciozyma antarctica*, both frequently recovered from persistently cold habitats^78,79^. *Glaciozyma*-related taxon isolates differed from *Glaciozyma antarctica* CBS 5842 by 27 nucleotides in the LSU marker and additionally contained a deletion of 5 nucleotides towards the end of the sequence, suggesting the possibility of a new taxon. *Glaciozyma* species are obligate psychrophiles, with a maximum growth temperature below 20 °C^78^ and found in cold habitats such as Alpine and Apennine glaciers^28,79^, Antarctic sea water and soil^81^, and Greenland glacial ice^81^. *Glaciozyma antarctica*, formerly *Leucosporidium antarcticum*^78^, has been widely studied due to its biotechnologically important production of antifreeze proteins and cold-active enzymes^83,84^. The genome of *Glaciozyma antarctica* was sequenced and analysed only recently^85^, revealing systems of psychrophilic response that are present only in this species and might be associated with temperature variations. The higher expression of such unique genes associated with cold adaptation unique to *G. antarctica*, together with other physiological strategies such as modelling its membrane lipid composition^86^, could explain the high abundance of the genus in cold environments and its superior ability, compared to other species, to overcome stressful conditions associated with extreme habitats. Genus *Phenoliferia* has been proposed by Wang *et al*.^79^ to accommodate a clade of species segregated from the genus *Rhodotorula*, characterised by the ability to assimilate phenol as the sole carbon source at 10 °C^87^. In particular *P. psychrophenolica* and *P. glacialis* can degrade up to 12.5 and 5 mM concentrations of phenol, respectively^86^. *P. psychrophenolica* has so far only been isolated from Alpine and Apennine glaciers^28,80,86^ and from the Gulkana Glacier in Alaska^25^ and the glacier Austre Broggerbreen on Svalbard^88^.

Additionally, many different yeasts species were isolated from our samples. These belonged mainly to genera *Rhodotorula*, *Mrakia*, *Filobasidium* and *Naganishia*. *Rhodotorula* is a ubiquitous genus commonly isolated from cold habitats^29^. *Rhodotorula* spp., belonging to the same class as *Glaciozyma* spp. (Microbotryomycetes), are also known to be part of the core yeast communities in cold environments^89^, with some species recovered exclusively from cold habitats and for this reason considered endemic and other species ubiquitous^89,90^. The ability to grow over a wide temperature range, its membrane fluidity stability, marked adaptability, nutritional versatility and polyextremotolerance^91,92^, together with the abundant production of photo-protective pigments^93^, make *Rhodotorula* spp. adept in withstanding extreme climates. *R*. *svalbardensis* is a novel psychrophilic species recovered from Svalbard glacier cryoconite holes^7^ and Greenland glacial ice^82^. We refer to the species as *R. svalbardensis* pro. tem. (*pro tempore*, temporary taxonomic placement) as suggested by Turchetti *et al*.^94^ due to its uncertain phylogenetic placement. Psychrophilic genus *Mrakia* has so far been restricted to the coldest areas of the world, such as Greenland, Antarctica, Siberia, Patagonia and Alaska^95,96^. The Principal Component Analysis based on cultivation data (Fig. 6A) revealed a strong correlation between the presence of the genera *Mrakia* and *Glaciozyma*-related taxon. Both yeasts were formerly classified as belonging to the *Leucosporidium* genus, characterised by an absence of visible carotenoid pigments production^97,98^. *Glaciozyma*-related taxon and *Mraki*a sp. (*gelida* or *frigida*) also share ecological traits: they are psychrophilic yeasts unable to grow at temperatures higher than 17 °C, with distribution consequently restricted to habitats with persistently low temperatures^97,98^. Further explanation of the co-occurrence of *Mrakia* and *Glaciozyma*-related taxon will be possible once the metabolic and other characteristics of the latter have been characterised further. *Filobasidium* species are widely spread, from very arid environments^99^ to Italian glacial meltwaters^100^ and basal ice of the High Arctic^4^. Species of the genus *Naganishia* are among the most resistant organisms to UV radiation^101^: they are found in many extreme environments such as high elevation soil of the Atacama Desert^101,102^, of the Antarctic Dry Valleys^81^, and of subglacial ice in Svalbard^4^.

Among cultivated filamentous fungi *Penicillium crustosum*, *P. bialowiezense*, and *Pseudogymnoascus* sp. prevailed. The frequent presence of *Penicillium spp*. in cold environments has been documented in various habitats, from soil to basal ice in the Arctic and Antarctic^6,103,104^. Sampling of subglacial ice of Pedersenbreen in 2001^6^ showed a high abundance of *P. crustosum* and this was confirmed in our study by cultivation and NGS from sample S-ice B1 originating from the same glacier. Also, amplicon sequencing showed a higher presence of *Penicillium* (1.3% of sequences) in this subsample compared to all other samples and glaciers (<0.2%). On the other hand, *Pseudogymnoascus* sp. was isolated exclusively from one glacier (Broggerbreen) (Fig. 6A), while no sequences belonging to this genus were recovered by culture-independent methods. Representatives of this cold-adapted genus were previously found in an alpine glacier of the Qinghai-Tibet Plateau^79^.

Other sporadically isolated filamentous fungi belonged to the genera *Lecanicillium*, *Thelebolus*, *Tetracladium*, *Leptosphaeria*, and *Mortierella*, all of which have previously been isolated from Antarctic soil samples^105^. *Mortierella* sp. was described as a decomposer of granite after glacier retreat^88^. Isolates in our study from S-ice B1 and G-wtr A closely related to *Venturia* (GenBank accession no. **AB916509**) were also isolated from feathers of a Barnacle goose on Svalbard^106^. Isolates of *Thelebolus* sp. (S-ice C1) closely related to *Thelebolus* strain with the GenBank accession no. **AB916508** were also isolated from bird feathers^106^. Genus *Thelebolus* has been described initially as an aquatic taxon from lake microbial mats in Eastern Antarctica^107^. Now it is recognized as one of the most common genera in polar soil crusts adjacent to glaciers^88,108^.

The overlap of the cultivation and amplicon sequencing results was modest, with only 8 out of 30 species found by cultivation also present in the amplicon sequencing results, again showing that each of the two approaches has its strengths and that cultivation and non-cultivation methods complement each other, as noted before by several authors investigating other environments^82^.

The presence of viable bacteria in subglacial ecosystems (deep glacier ice and subglacial waters, basal ice, subglacial sediments and lakes) has been intensively studied for several years^13,16,18,109–112^. Subglacial habitats are characterized by higher cells numbers compared to englacial habitats and can reach abundances up 10^5^ cells/ml^113^. Canadian subglacial meltwater samples yielded more than 10^3^ CFU/ml^17^ or 100-fold more than our samples (up to 10^3^ CFU/100 ml).

In line with previous studies^13,114,115^ culture independent results identified Proteobacteria and Actinobacteria as the most abundant phyla in almost all samples. On the one hand the composition of the bacterial populations varied between samples from the same glacier, but were on the other hand similar between some sample pairs collected from different glaciers. For example, subglacial ice samples S-ice-A1 (Lovenbreen) and S-ice-B2 (Pedersenbreen) were both dominated by *Sulfuricurvum* sp., a novel genus isolated from an underground crude-oil storage cavit^116^. *Sulfuricurvum* is a facultatively anaerobic, chemolithoautotrophic bacterium previously isolated from subglacial lakes in Iceland^117,118^. It uses elemental sulphur, sulphide and thiosulfate as electron donors during microaerobic (1% O_2_) and anaerobic respiration^118^. Sulphur is common in subglacial bedrocks, favoring microbial sulphide oxidation, and sulphate reduction^19^. Thus *Sulfuricurvum* spp. might play a role in the biogeochemical cycling of sulphur in the subglacial ice, which is in direct contact with the underlying bedrock.

While the fungal diversity of different glacial samples differed considerably even between samples from the same glacier, the differences in bacterial diversity was much smaller, with *Micrococcus* (Actinobacteria) being the most abundant species in all samples. *Micrococcus* has been previously found in ice, for example in 20.000 years old Bolivian core ice samples^119^ and Malan Glacier ice cores^120^.

Cultivation dependent results for bacteria overlapped to some extent with the results of the amplicon sequencing: 17 species of bacteria were isolated, 12 of them belonging to the phylum Proteobacteria. However, the most commonly isolated species was *Flavobacterium* sp. (Bacteroidetes), which represented less than 4% of sequenced amplicons. Members of this genus are commonly isolated from aquatic ecosystems^121^. *Flavobacterium* species are considered psychrotolerant, since they have been isolated from several polar habitats including Antarctic soil, lakes, marine sediment and sea ice^122–125^, high Artic snow cover^1^, and frozen soil in China^126^. The genus contains the causative agent of bacterial cold water disease, *F. psychrophilum*^127^. Among other isolated bacterial species were *Massilia* sp. and *Polaromonas* sp. (Oxalobacteraceae and Comamonadaceae families, respectively). *Massilia* members were originally isolated from clinical samples^128^ and characterized as aerobic and facultatively psychrophilic^129^. Later they were found in various habitats such as freshwater^130^, aerosols, air, and house dust^131–133^, soil^134,135^, and phyllosphere^136,137^ and also from glacier ice cores in China from which two novel species, *M. eurypsychrophila* and *M. glaciei*, were recently described^138,139^. Cold-active bacteria of the genus *Polaromonas* (class Betaproteobacteria) have also been recognised as significant components of glacial microbiomes before^140^.

## Conclusions

We investigated the microbial diversity of subglacial ice and nearby aquatic environments of three polythermal Arctic glaciers located in the Kongsfjorden area on Svalbard. The main findings of the study are:

- Subglacial ice of polythermal glaciers harbours a rich and spatially heterogenous fungal and bacterial diversity.
- Several as yet undescribed species were isolated, including strains related to the genera *Glaciozyma* and *Rhodotorula*.
- The first amplicon sequencing of the subglacial ice mycobiota uncovered species previously not reported from the Arctic ecosystems, such as the extremely halotolerant *Hortaea werneckii* and the skin pathogen *Malassezia restricta*.
- Of additional interest was the high abundance of the elusive phylum Chytridiomycota and their parasites Rozellomycota in the snow-derived tap water, but of Chytridiomycota also (and even more surprisingly) in the glacial ice.

In summary, the first study combining the cultivation and amplicon sequencing to investigate the diversity of both fungi and bacteria in subglacial ice of polythermal glaciers has shown a rich diversity of species, an unexpected spatial heterogeneity of (particularly fungal) communities and has found several species that are either new to science or have never been found in glacial ice before.

## Materials and Methods

### Site and samples description

Fieldwork was conducted during the end of July and beginning of August 2017 in the Kongsfjorden area (Ny-Ålesund) of the Svalbard Archipelago, located at 79°N, 12°E, Norway. Subglacial ice, clear ice, sea water, lake water and glacial meltwater samples were collected from the locations shown in Table 1 and Fig. 1. Subglacial ice and clear ice floating on seawater were sampled by chopping the ice from the glacier edges or from the floating growlers (smaller fragments of ice) with surface-sterilized tools and transferred into sterile *Whirl-Pak*® plastic bags. Glacial melt-water, moraine lake water, seawater and tap water were directly collected into sterile plastic bottles. All samples were transported to the lab for preparation within five hours of collection and processed at the NERC Arctic Research Station, Ny-Ålesund. Glaciers studied are small valley glaciers ending on land with similar surface areas (5–5.6 km^2^): Midtre Lovénbreen, Pedersenbreen, and Vestre Brøggerbreen. They are all non-temperate valley glaciers with polythermal characteristics^141^, and subject to substantial glaciological and ecological studies^142,143^. The glacier thermal regime is important in defining the presence of water at its bed and consequently exerts a firm influence on the subglacial drainage system development^12^.

**Table 1:** Characteristics of samples and GPS coordinates of sampling sites. Locations are shown in Fig. 1.

Site	Midtre Lovénbreen				Pedersenbreen		Vestre Brøggerbreen		Kongsfjorden				UK NERC station, Ny-Ålesund
Name samples - amplicon seq.	**S-ice A1**	**S-ice A2**	**G-wtr A**	**L-wtr**	**S-ice B1**	**S-ice B2**	**S-ice C1**	**S-ice C2**	**C-ice 1**	**C-ice 2**	**Sea H**	**Sea F**	**Tap**
Site latitude (decimal)	78.893572	78.893944	78.893535	78.900106	78.879527	78.879616	78.915354	78.915316	78.886029	78.886029	78.928113	78.937911	78.92534
Site longitude (decimal)	12.070259	12.069138	12.070333	12.069259	12.299257	12.303359	11.763458	11.761196	12.436453	12.436453	11.935260	11.937170	11.93239
N° of samples	4				2		2		4				1
Type of samples	subglacial ice		glacial meltwater	moraine lake water	subglacial ice		subglacial ice		clear glacial ice		sea water harbour	sea water fjord	tap water
Cultivation	+	+	+		+	+	+	+					
Volume filtered - amplicon seq.	200 ml	750 ml	400 ml	1.5 L	750 ml	500 ml	500 ml	250 ml	500 ml	1 L	1 L	1 L	3 L

### Cultivation-based fungal and bacterial diversity analyses

The surface layer of subglacial ice samples was melted in a sterile container at room temperature and discarded. The remaining ice was washed with sterile water, transferred to another sterile container and melted. Five, 20 and 50 ml aliquots of subglacial ice samples and glacial meltwater were filtered through Milli-pore membrane filters (0.45 μm pore size) in duplicate. Due to the high sediment content of the subglacial ice and glacial meltwater, the samples were vortexed and left to sediment for one minute before filtering. Filters were placed on two enumeration and four different selective agar media with either low water activity (a_w_) or low nutrient content. Since fungal diversity is usually neglected, greater effort in fungal isolation was attempted. The media used were therefore DRBC - a general-purpose enumeration medium^144^; MY10–12 - a medium for the isolation of xero- and halo-tolerant fungi with 10% glucose and 12% NaCl (a_w_ = 0.880)^145^; DG-18 - a medium for detection of moderate xerophiles (a_w_ = 0.946)^146^; SNA and MM – two nutrient-poor media for the isolation of oligotrophic fungi and R2A - a low nutrient enumeration medium for heterotrophic microorganisms (both bacteria and fungi)^147^. To prevent the growth of fast-growing prokaryotes, chloramphenicol (50 mg/l) was added to all media, except for R2A agar. Plates were incubated at 10 °C for up to 8 weeks at the University of Ljubljana, where subsequent procedures were carried out. For every medium the average number of colony forming units (CFU/100 ml) was counted and calculated.

### Fungal and bacterial identification

DNA was extracted from pure fungal and bacterial cultures up to one week after incubation on a malt extract agar (MEA) and R2A media, respectively. DNA from filamentous fungi was extracted by mechanical lysis of approx. 1 cm^2^ of mycelium according to Van den Ende and de Hoog (1999)^148^. For yeast-like and bacterial strains, DNA was extracted using PrepMan Ultra reagent (Applied Biosystems) according to the manufacturer instructions. For filamentous fungi a fragment of rDNA including ITS region 1, 5.8S rDNA and ITS region 2 (referred from here on as the ITS amplicon) was amplified using ITS5 and ITS4 primers^149^. Polymerase chain reactions (PCR) were performed using Thermo Scientific *Taq* DNA Polymerase according to the manufacturer’s protocol. Reactions were run in a PCR Mastercycler Ep Gradient (Eppendorf) with an initial denaturation of 2 min at 95 °C, followed by 30 cycles of denaturation at 95 °C for 45 s, annealing at 54 °C for 30 s, and elongation at 72 °C for 2 min, with a final elongation at 72 °C for 4 min. For identification of *Penicillium* spp. strains, the partial β-tubulin gene (benA) was amplified and sequenced with Ben2f and Bt2b primers^150^. Initial denaturation at 95 °C for 1 min was followed by 35 cycles of denaturation at 95 °C for 30 s, annealing at 53 °C for 30 s, and elongation at 72 °C for 1 min. Final elongation was at 72 °C for 10 min. *Cladosporium* strains were identified using partial actin (act) sequences, amplified with ACT-512F and ACT-783R primers^151^. Initial denaturation at 94 °C for 5 min was followed by 45 cycles of denaturation at 94 °C for 45 s, annealing at 52 °C for 30 s and elongation at 72 °C for 90 s. Final elongation was at 72 °C for 5 min. For yeasts domains D1 and D2 of LSU rDNA gene were amplified using NL1 and NL4 primers^152^. Initial denaturation at 95 °C for 5 min was followed by 30 cycles of denaturation at 95 °C for 45 s, annealing at 54 °C for 30 s and elongation at 72 °C for 2 min. Final elongation was at 72 °C for 4 min. For bacteria 16S rRNA gene was amplified with 27f-lane and 1492 R primers^153^ and a touchdown program. Initial denaturation at 95 °C for 5 min was followed by 5 cycles of denaturation at 95 °C for 30 s, annealing at 60 °C for 30 s and elongation at 72 °C for 1 min; 5 cycles of denaturation at 95 °C for 30 s, annealing at 55 °C for 30 s and elongation at 72 °C for 1 min; and 30 cycles of denaturation at 95 °C for 30 s, annealing at 50 °C for 30 s and elongation at 72 °C for 1 min. Final elongation was at 72 °C for 7 min. The ITS, LSU, benA, act and 16S nucleotide sequences were determined by Sanger sequencing, performed by Microsynth AG, Switzerland. The resulting sequences of all the isolates were aligned using MUSCLE software^154^ implemented in MEGA5 package^155^ and compared against the GenBank database using the BLAST software^156^. Maximum likelihood methods implemented in PhyML 3.0^157^ were used to build phylogenetic trees after aligning the sequences with similar type and reference sequences from the GenBank database in MEGA5^155^ (Supplementary Material). Bootstrap values were calculated from 500 replicate runs. A principal component analysis (PCA) based on presence/absence data of fungal species in the studied environments was performed to determine which species best explained the variance among samples. The PCA was performed in R with the *prcomp* function^158^ and plotted with *ggbiplot*^159^.

All isolated strains have been deposited in the Ex Culture Collection of the Infrastructural Centre Mycosmo (MRIC UL) at the Department of Biology, Biotechnical Faculty, University of Ljubljana, Slovenia.

### Fungal and bacterial diversity analysis with amplicon sequencing

DNA was extracted from filtered biomass (0.45 μm pore size, Millipore) of subglacial ice (S-ice), clear ice (C-ice), sea water (Sea), glacial meltwater (G-wtr) and moraine water (L-wtr). All filters were placed in 1.5 ml microcentrifuge tubes containing RNAlater® (Sigma-Aldrich Company Ltd., UK) and immediately frozen at −20 °C, until further analysis in the laboratory setting. DNA was extracted from filters using the PowerWater DNA Isolation Kit (MoBio Laboratories Inc., California) and then from the same filters using the PowerLyzer PowerSoil DNA Isolation Kit (MoBio), according to the manufacturer’s instructions with a slight modification to increase the DNA yield and quality. To increase efficiency of fungal cells lysis an additional heating incubation at 65 °C for 10 min was used after adding PW1 solution. DNA from both isolation methods was pooled together and stored at −80 °C until PCRs were performed.

For the analysis of fungal diversity, Illumina Miseq V3 (300 bp paired-end, performed by Microsynth AG, Switzerland) sequencing was carried out on the ITS2 region of the ITS rDNA gene amplified using the primers ITS4-Fun (5′- AGCCTCCGCTTATTGATATGCTTAART -3′) and 5.8S-Fun (5′- AACTTTYRRCAAYGGATCWCT -3′)^160^. Amplification was carried out in a PCR Mastercycler Ep Gradient (Eppendorf) with initial denaturation of 2 min at 98 °C, followed by 20 cycles of 10 s at 98 °C, 25 s at 54 °C and of 25 s at 72 °C, with a final elongation of 7 min at 72 °C.

For the analysis of bacterial diversity, Illumina Miseq V2 (250 bp paired-end, performed by Microsynth AG, Switzerland) sequencing was carried out on the hypervariable V3 and V4 regions of the 16S rRNA gene by using the 341F_ill (5′- CCTACGGGNGGCWGCAG -3′) and 802R_ill (5′- GACTACHVGGGTATCTAATCC -3′) universal bacterial primers^161^.

For both fungi and bacteria, the first step polymerase chain reactions (PCR) were performed in house using the Phusion® High-Fidelity DNA Polymerase according to manufacturer’s protocol, due to the low amount of DNA. Reactions were run in a PCR Mastercycler Ep Gradient (Eppendorf) with initial denaturation of 3 min at 95 °C, followed by 20 cycles of 20 s at 95 °C, 30 s at 56 °C and of 30 s at 72 °C, with a final elongation of 5 min at 72 °C. S-ice-C2 was the only sample from which no amplicons (either ITS or 16S) could be amplified, despite several attempts and optimisation of the amplification protocol. The latter sample was therefore excluded from the amplicon sequencing analyses.

Paired-end reads were quality checked and trimmed (minimum quality score 20) and analyzed with QIIME2 2018.8 software package (Quantitative Insights Into Microbial Ecology)^162^. Since the assembly of the paired end reads was largely unsuccessful, only single-end forward reads were used in the subsequent analysis. The reads were denoised, the tree was constructed by FastTree on a mafft alignment and rooted at midpoint and the alpha and beta diversity indices were calculated. For assigning the taxonomy to sequences, the 99% cut-off GreenGene database^163^ was used for training the feature classifier for bacteria, and the dynamically clustered UNITE ITS database^164^ was used for fungi. Abundances in each sample were normalized to the number of sequences in the least abundant sample. Due to the high amount of amplified chloroplast DNA in lake water samples and in order to investigate the bacterial diversity in all samples in a comparable way, chloroplast sequences were excluded with a taxonomy-based filtering. Shannon index was calculated to study the alpha diversity. The distance and dissimilarity matrices were determined through Unifrac distances to visualize the ordination and clustering of the bacterial and fungal community composition for beta diversity analyses. The ordination patterns based on phylogenetic distance metrics were evaluated using principal coordinate analysis (PCoA). Differences in microbial community composition between samples types were assessed by non-parametric permutational analysis of variance (PERMANOVA). All analyses above were performed in QIIME2 2018.8 software package (Quantitative Insights Into Microbial Ecology)^162^. To evaluate community similarities between samples, a hierarchical clustering analysis of the taxa abundance in the communities was performed with the *pheatmap* and *hclust* packages in R^158,165^.

### Sequences

Bacteria GenBank MK670504-MK670553.

Yeasts GenBank MK670448-MK670503.

Filamentous fungi GenBank MK671591-MK671647.

NGS fungi GenBank PRJNA 517070.

NGS bacteria GenBank PRJNA 517069.

## Supplementary information

Supplementary Dataset 1

## Data Availability

The data generated during the current study are available in the GenBank repository, and are included in this published article (and its Supplementary Information files).
